# Varnishing Cavities

**Published:** 1897-07

**Authors:** W. G. Browne

**Affiliations:** Atlanta, Ga.


ARTICLE VI.

### VARNISHING CAVITIES.

BY DR. W. G. BROWNE, ATLANTA, GA.

The incompatibility of tooth-substance and the metals we use for filling teeth is a well-recognized fact, and it is always good practice to interpose some substance between the metal and tooth-structure to prevent, as far as possible any injurious effect from such incompatibility. Gutta-percha, chloro-percha, cement, and varnish, each have their merits, but none seem to have so many points of excellence as a clear resin, such as damson, dissolved in chloroform. It acts as a non-conductor of thermal changes as well as an insulator against electrical influences. It is not readily soluble in the fluids of the mouth. Being transparent, no discoloration is shown when used, where enamel walls are thin; in fact, it prevents discoloration of the tooth from oxidation when an amalgam is used which contains metals which oxidize in the mouth. To a limited degree, it may act as a support to frail walls of enamel, especially if the filling be inserted while the varnish is in a plastic state; this refers more especially to amalgam fillings.

In the insertion of large gold fillings it is helpful in starting the filling, holding the first mats or cylinders of gold firmly adherent to the dentine, and makes it almost out of the question for gold fillings to come out if proper attention has been given to the method of applying the varnish and gold in the teeth when commencing the filling.

I do not for a moment advance the idea that we should depend on the varnish to retain the filling, independent of other means, but it will not be found necessary to make deep retaining pits, but only slight undercuts in the most convenient places in the cavity, thus saving the operator valuable time, and no harm can possibly result from its use, while much good must come.

I am satisfied that when the profession realizes the benefit accruing from this method it will be universally adopted.—*Southern Journal.*

---

ARTICLE VII.

### AN APPARATUS FOR DRAINAGE.

